# Serum p53 autoantibodies in patients with minimal lesions of ductal carcinoma in situ of the breast

**DOI:** 10.1038/sj.bjc.6690751

**Published:** 1999-10

**Authors:** S Regele, P Kohlberger, F D Vogl, W Böhm, R Kreienberg, I B Runnebaum

**Affiliations:** Department of Obstetrics and Gynaecology, University of Ulm, Prittwitzstr. 43, Ulm, 89075, Germany; Department of Obstetrics and Gynaecology, University of Freiburg, Hugstetterstr. 55, Freiburg, 79106, Germany

**Keywords:** breast cancer, ductal carcinoma in situ, p53, autoantibodies

## Abstract

Five of 43 patients (11.6%) with ductal carcinoma in situ of the breast presented with p53 autoantibodies at diagnosis. Three seropositive patients demonstrated tumour sizes of ≤ 5 mm. There was no association of p53 autoantibody status with age, clinical presentation, histological subtype, tumour size, grading, p53 immunohistochemistry or hormone receptor status. © 1999 Cancer Research Campaign


*British Journal of Cancer* (1999) **81**(4), 702–704
© 1999 Cancer Research Campaign
Article no. bjoc.1999.0751

### Serum p53 autoantibodies in patients with minimal lesions of ductal carcinoma in situ of the breast

**S Regele[1], P Kohlberger[2], FD Vogl[1], W Böhm[1], R Kreienberg[1] and IB Runnebaum[2]**

[1]University of Ulm, Department of Obstetrics and Gynaecology, Prittwitzstr. 43, 89075 Ulm, Germany; [2]University of Freiburg, Hugstetterstr. 55, Department of Obstetrics and Gynaecology, 79106 Freiburg, Germany

**Summary** Five of 43 patients (11.6%) with ductal carcinoma in situ of the breast presented with p53 autoantibodies at diagnosis. Three seropositive patients demonstrated tumour sizes of ≤ 5 mm. There was no association of p53 autoantibody status with age, clinical presentation, histological subtype, tumour size, grading, p53 immunohistochemistry or hormone receptor status. © 1999 Cancer Research Campaign

**Keywords:** breast cancer; ductal carcinoma in situ; p53; autoantibodies

Mutation of the p53 tumour suppressor gene has been reported in 20–40% of invasive breast cancer (Runnebaum et al, 1991; Coles et al, 1992; Sjogren et al, 1996). Mutant p53 protein accumulates in the nucleus of neoplastic cells. Autoantibodies against p53 protein (p53 AAb) were detected in sera of breast cancer patients in 5–26% (Schlichtholz et al, 1992; Angelopoulou et al, 1994; Mudenda et al, 1994; Peyrat et al, 1995). In invasive breast cancer, the presence of p53 AAb is associated with parameters of poor prognosis such as high nuclear grade and absence of hormone receptors (Mudenda et al, 1994; Peyrat et al, 1995). For patients with breast, colorectal and lung tumours, the specificity of p53 AAb for malignancy is close to 100% (Crawford et al, 1982; Winter et al, 1992; Angelopoulou et al, 1997).

Ductal carcinomas in situ (DCIS) of the breast represent about 20% of all breast carcinomas diagnosed by mammographic screening (Schnitt et al, 1988). Nearly 95% of DCIS are detected because of microcalcifications (Peeters et al, 1989). A palpable breast lump develops when there is fibrotic tissue around the DCIS lesion (Silverstein, 1997). Poorly differentiated DCIS often exhibit branching or coarse-granular microcalcifications. Fine granular microcalcifications are associated with well differentiated DCIS, but they appear similar to microcalcifications of benign lesions (Holland and Hendriks, 1994). A simple serum test with a high specificity for malignancy could help to decide about invasive diagnostic steps. The objective was to determine the prevalence of p53 AAb in preoperative sera of patients with DCIS and to relate p53 AAb status with age, clinical presentation, DCIS subtype, tumour size, grading, p53 immunohistochemisry (IHC) and hormone receptor status.

### PATIENTS AND METHODS

### Patients

From April 1995 to October 1996, 53 subsequent patients underwent at least excisional biopsy for DCIS at the Department of Obstetrics and Gynaecology, University of Ulm. Serum of 43 patients with histologically diagnosed DCIS of the breast was available for analysis. Sera were collected preoperatively and were stored at –80°C. By routine mammography 26 patients had suspicious areas with granular microcalcifications, one patient demonstrated a suspicious dense lesion. Thirteen patients presented with a palpable breast lump and three patients complained of nipple discharge. Two patients had previously been treated for invasive breast cancer of the contralateral breast. The median age of the patients was 52 years ranging from 26 to 86 years.

*Received 2 October 1998*
*Revised 14 April 1999*
*Accepted 22 April 1999*

*Correspondence to:* IB Runnebaum

### Serological analysis p53 AAb were measured in serum using a sandwich enzyme-linked immunosorbent assay (ELISA) with microtitre plates coated with recombinant human p53 protein expressed in *Escherichia coli* (Dianova, Hamburg). Compared to control sera supplied by the manufacturer, results were categorized as negative, low, medium and high level.

### Histology

Formalin-fixed, paraffin-embedded tissue samples from all 43 patients were analysed. Nuclear grade was defined as grades 1–3 according to previously described criteria (Lagios, 1990). The histological subtype was classified in comedo and noncomedo subtype.

### Immunohistochemistry

Tumour cell staining for p53 protein was performed with the monoclonal antibody DO-1 (Dianova, Hamburg). Immunostaining for oestrogen (ER) and progesterone receptors (PR) was performed with the monoclonal antibodies ID-5 (Immunotech, Hamburg) and 1A-6 (Dako, Hamburg) respectively. For semiquantitative assessment, immunohistochemical staining for nuclear p53 of more than 10% of the tumour cells was interpreted as positive. Hormone receptor status was scored according to an immunoreactive score assessing the proportion of stained cells and the staining intensity (Remmele and Stegner, 1987).

**Table 1** Clinical and histopathological parameters with regard to p53 AAb status

| | p53 AAb-positive | |
|---|---|---|
| **Parameters** | ***n*** | **(%)** |
| Age | | |
| < 50 years | 3/21 | (14.3%) |
| ≥ 50 years | 2/22 | (9.1%) |
| Clinical presentation | | |
| Palpable lump | 2/13 | (15.4%) |
| Microcalcifications | 3/27 | (11.1%) |
| Nipple discharge | 0/3 | (0%) |
| p53 Immunohistochemistry | | |
| Positive | 3/11 | (27.3%) |
| Negative | 2/19 | (27.7%) |
| Hormone receptor status | | |
| ER positive | 3/19 | (15.8%) |
| ER negative | 2/10 | (20.0%) |
| PR positive | 3/14 | (21.4%) |
| PR negative | 2/13 | (15.4%) |
| Histological grading | | |
| G1 | 0/1 | (0%) |
| G2 | 4/18 | (22.2%) |
| G3 | 1/2 | (50.0%) |
| Histological subtype of DCIS | | |
| Comedo | 1/9 | (11.0%) |
| Non-comedo | 4/19 | (21.1%) |
| Tumour size | | |
| ≤ 10 mm | 3/10 | (30.0%) |
| > 10 mm | 1/13 | (7.7%) |
| Disseminated lesions | 1/20 | (5.0%) |

### RESULTS

p53 AAb were detected in serum of 5/43 patients (11.6%) with DCIS, three of which had lesion sizes ≤ 5 mm. p53 AAb titre was low in all five sera compared with assay control samples. The two patients previously treated for invasive breast cancer had negative test results in the ELISA. p53 AAb status with regard to clinical and histopathological parameters are presented in Table 1. Tumour samples were evaluable for immunohistochemistry from 30/43 patients due to the small size of the tumour tissue. Expression of ER and PR was analysed in 29 and 27 patients respectively. In 23 cases of non-disseminated lesions, the size ranged from 3 to 90 mm (mean 21 mm). In the 20 patients with disseminated DCIS lesions the tumour size could not be exactly determined. Nuclear grade could be assessed in 21/43 tumours due to the heterogeneity of the tissue. Clinical presentation and histopathological data of p53 AAb-positive patients are listed in Table 2. No association of p53 AAb status with age, clinical presentation, tumour size, grading, p53-IHC, hormone receptor status and histological subtype was observed. Only one of nine patients with comedo subtype DCIS was positive for p53 AAb with > 90% p53-positive tumour cells.

### DISCUSSION

The prevalence of p53 AAb in our cohort of patients with DCIS (11.6%) was comparable to the prevalence found in invasive breast cancer which varied from 5% to 26% (Schlichtholz et al, 1992; Angelopoulou et al, 1994; Mudenda et al, 1994; Peyrat et al, 1995). In a population-based study ($n$ = 237) we found an overall prevalence of 16.5% in patients with invasive breast cancer using the same test system as in this study (unpublished data). In a previous study applying an ELISA technique and testing 182 newly diagnosed breast cancer patients, 8/23 (35%) patients with DCIS had p53 AAb (Mudenda et al, 1994). Our ELISA assay was tested on 41 healthy blood donors in another study, all of which were negative for p53 AAb (Maass et al, 1996). This demonstrates the high specificity for malignancy. The variation in the reported p53 AAb prevalences may be explained by the different techniques applied such as ELISA, immunoprecipitation or immunoblot (Angelopoulou et al, 1994; Mudenda et al, 1994; Peyrat et al, 1995). It may also be due to different clinical stages within the study populations. Interestingly, in our study p53 AAb were detected in patients with tumour lesions of 3 mm and 5 mm. Therefore, p53 AAb immunogenicity of breast cancer cells was not associated with extended lesions. Two patients with p53 AAb had no p53 protein expression in the tumour. This observation was also made in a previous study (Mudenda et al, 1994) with five of 23 seropositive breast cancer patients who had no detectable p53 accumulation in the tumour. In these patients, the immunogenic reaction may have occurred earlier in tumour development or factors other than accumulation such as the type of p53 mutation or complex formation between p53 and a 70 kDa heat shock protein contributed to the p53 AAb production (Davidoff et al, 1992). p53 AAb can already be present in patients with early, non-palpable lesions. Given the high specificity for malignancy, the p53 AAb ELISA may become a helpful tool in the diagnostic process in subsets of DCIS patients to be defined in further studies.

### ACKNOWLEDGEMENT

The authors thank Tanja Köhler for excellent technical assistance. The study was supported by the Deutsche Forschungsgemeinschaft (Ru 476/2-2) granted to IBR.

**Table 2** Clinical and histopathological data of p53 AAb-positive patients

| No. | Clinical presentation | Tumour size | Grading | Subtype | p53 IHC | ER/PR |
|---|---|---|---|---|---|---|
| 1 | Palpable lump | 3 mm | G2 | Non-comedo | positive[a] | positive[b] |
| 2 | Microcalcifications | 5 mm | G2 | Non-comedo | positive | positive |
| 3 | Microcalcifications | 3 mm | G2 | Non-comedo | negative | positive |
| 4 | Palpable lump | 25 mm | G2 | Non-comedo | negative | negative |
| 5 | Microcalcifications | Disseminated small lesions | G3 | Comedo | positive | negative |

[a]More than 10% positive tumour cells in IHC. [b]According to immunoreactive score (Remmele and Stegner, 1987).

© 1999 Cancer Research Campaign 

### REFERENCES

Angelopoulou K, Diamandis EP, Sutherland DJ, Kellen JA and Bunting PS (1994) Prevalence of serum antibodies against the p53 tumor suppressor gene protein in various cancers. *Int J Cancer* **58**: 480–487

Angelopoulou K, Stratis M and Diamandis EP (1997) Humoral immune response against p53 protein in patients with colorectal carcinoma. *Int J Cancer* **70**: 46–51

Coles C, Condie A, Chetty U, Steel CM, Evans HJ and Prosser J (1992) p53 mutations in breast cancer. *Cancer Res* **52**: 5291–5298

Crawford LV, Pim DC and Bulbrook RD (1982) Detection of antibodies against the cellular protein p53 in sera from patients with breast cancer. *Int J Cancer* **30**: 403–408

Davidoff AM, Iglehart JD and Marks JR (1992) Immune response to p53 is dependent upon p53/HSP70 complexes in breast cancers. *Proc Natl Acad Sci USA* **89**: 3439–3442

Holland R and Hendriks JH (1994) Microcalcifications associated with ductal carcinoma in situ: mammographic-pathologic correlation. *Semin Diagn Pathol* **11**: 181–192

Lagios MD (1990) Duct carcinoma in situ. Pathology and treatment. *Surg Clin North Am* **70**: 853–871

Maass JD, Gottschlich S, Lippert BM, Niemann AM, Gorogh T and Werner JA (1996) Antibody formation to cellular p53 protein in patients with squamous cell carcinomas of the upper respiratory and digestive system. *Laryngorhinootologie* **75**: 53–56

Mudenda B, Green JA, Green B, Jenkins JR, Robertson L, Tarunina M and Leinster SJ (1994) The relationship between serum p53 autoantibodies and characteristics of human breast cancer. *Br J Cancer* **69**: 1115–1119

Peeters PH, Verbeek AL, Hendriks JH and van Bon MJ (1989) Screening for breast cancer in Nijmegen. Report of 6 screening rounds, 1975–1986. *Int J Cancer* **43**: 226–230

Peyrat JP, Bonneterre J, Lubin R, Vanlemmens L, Fournier J and Soussi T (1995) Prognostic significance of circulating p53 antibodies in patients undergoing surgery for locoregional breast cancer. *Lancet* **345**: 621–622

Remmele W and Stegner HE (1987) [Recommendation for uniform definition of an immunoreactive score (IRS) for immunohistochemical estrogen receptor detection (ER-ICA) in breast cancer tissue]. *Pathologe* **8**: 138–140

Runnebaum IB, Nagarajan M, Bowman M, Soto D and Sukumar S (1991) Mutations in p53 as potential molecular markers for human breast cancer. *Proc Natl Acad Sci USA* **88**: 10657–10661

Schlichtholz B, Legros Y, Gillet D, Gaillard C, Marty M, Lane D, Calvo F and Soussi T (1992) The immune response to p53 in breast cancer patients is directed against immunodominant epitopes unrelated to the mutational hot spot. *Cancer Res* **52**: 6380–6384

Schnitt SJ, Silen W, Sadowsky NL, Connolly JL and Harris JR (1988) Ductal carcinoma in situ (intraductal carcinoma) of the breast. *N Engl J Med* **318**: 898–903

Silverstein MJ (1997) *Ductal Carcinoma In Situ of the Breast*. Williams and Wilkins: Baltimore

Sjogren S, Inganas M, Norberg T, Lindgren A, Nordgren H, Holmberg L and Bergh J (1996) The p53 gene in breast cancer: prognostic value of complementary DNA sequencing versus immunohistochemistry. *J Natl Cancer Inst* **88**: 173–182

Winter SF, Minna JD, Johnson BE, Takahashi T, Gazdar AF and Carbone DP (1992) Development of antibodies against p53 in lung cancer patients appears to be dependent on the type of p53 mutation. *Cancer Res* **52**: 4168–4174

© 1999 Cancer Research Campaign
